# HR-LCMS/MS-Based Dereplication of Plant-Derived Autophagy Inducers Revealed *Astragalus dasyanthus* as a New Glabrol Producer

**DOI:** 10.3390/metabo16050311

**Published:** 2026-05-01

**Authors:** Anastasiia K. Bolikhova, Vera A. Alferova, Anton R. Izzi, Arina A. Nikandrova, Gulnara K. Kudryakova, Mikhail Y. Zhitlov, Ekaterina A. Guseva, Dmitry S. Karpov, Dmitrii A. Lukianov, Olga A. Dontsova, Petr V. Sergiev

**Affiliations:** 1 Center for Bio- and Medical Technologies, 121205 Moscow, Russia; 2 Belozersky Institute of Physico-Chemical Biology, Lomonosov Moscow State University, 119991 Moscow, Russia; 3 Department of Chemistry, Lomonosov Moscow State University, 119991 Moscow, Russia; 4 Shemyakin-Ovchinnikov Institute of Bioorganic Chemistry, Russian Academy of Science, 117997 Moscow, Russia; 5 Faculty of Bioengineering and Bioinformatics, Lomonosov Moscow State University, 119991 Moscow, Russia; 6 Engelhardt Institute of Molecular Biology, Russian Academy of Sciences, 119991 Moscow, Russia

**Keywords:** plant extracts, novel resource of autophagy inducers, LC3 I/II, glabrol, *Astragalus dasyanthus*

## Abstract

Background/Objectives: Autophagy is an important cellular self-cleansing process whose normal functioning is essential for preventing many age-related diseases. The search for and study of new autophagy activators allows the proposal of novel therapeutic approaches for the treatment of age-related diseases. Medical plants are a rich source of bioactive compounds with variable functions. In this study, we propose an HR-LCMS/MS-based technique for identifying the principal autophagy activators in plant extracts. Methods: We performed a Western blot analysis of the autophagy-inducing activity of plant extract HPLC fractions on a model of SH-SY5Y cells. The composition of the fractions showing autophagy-activating potential was determined via HR-LCMS/MS. Results: We analyzed five plants known to produce autophagy activators and proved the ability of the method to detect the main bioactive compounds. Additional screening demonstrated for the first time that *Astragalus dasyanthus* is a producer of the autophagy-inducer glabrol. Conclusions: The described HR-LCMS/MS-based method for identifying autophagy activators in multicomponent plant extracts is effective and could be used for further high-throughput screening.

## 1. Introduction

Autophagy is an essential mechanism of cellular “self-cleaning”, responsible for the removal of defective cellular components and the maintenance of intracellular homeostasis; proper activation of autophagy is crucial for sustaining numerous cellular processes [1,2]. For example, mitophagy, a specific type of autophagy involving the removal of damaged mitochondria, is necessary for maintaining normal energy metabolism and preventing oxidative stress [3,4]. At the same time, the degradation of misfolded proteins helps to prevent the development of neurodegenerative diseases [5,6]. Therefore, the search for compounds capable of activating autophagy is a highly promising direction for treating and alleviating numerous diseases, including diabetes [7], atherosclerosis [8], Alzheimer’s, and Parkinson’s diseases [9,10].

Various plants often serve as sources of autophagy inducers [11,12]. Plants have been used in traditional medicine for centuries; however, the mechanisms underlying their effects remained unknown for a long time. Currently, increasing attention is being paid to the precise composition of medicinal herbs and the development of new approaches for utilizing bioactive components within them [13,14].

One of the main approaches for the identification of bioactive compounds is chromatographic separation of plant extracts followed by mass spectrometry analysis of each fraction (LS/MS). This approach enables the construction of a chemical profile of the given extract [13,15,16]. Despite the fact that for many plant species, a comprehensive set of metabolites has already been described, the phytochemical composition of a plant extract depends on the plant part used, the stage of the life cycle, the season, or even the region of collection [17,18,19]. The rapid identification of active compounds across many samples is therefore a critical task.

In this study, we describe a simplified method for fast identification of major autophagy inducers from natural sources using HR-LCMS/MS. The human neuroblastoma cell line SH-SY5Y was selected as a model system, as it is frequently used in autophagy research and allows for the assessment of the potential neuroprotective effects of candidate compounds. A panel of ethanolic extracts from a variety of herbs united by their use in traditional medicine in different countries was prepared; for some of these plants, the presence of significant amounts of autophagy-inducing compounds had been previously reported, while for others it had not. The ability of the extracts to induce autophagy in SH-SY5Y cells was assessed by Western blot analysis using antibodies against LC3 I/II. Active extracts were subsequently subjected to chromatographic fractionation (HPLC). Using the same Western blot approach to identify active fractions and based on mass spectrometry data, dereplication was performed to determine the principal compound responsible for the autophagic effect of each extract. As a result, for five plants known to produce autophagy activators, the main bioactive compound was detected, showing the applicability of the method. In the course of additional screening, *Astragalus dasyanthus* was identified as a new source of the autophagy inducer glabrol.

## 2. Materials and Methods

### 2.1. Ethanolic Extraction

The plant material was acquired from the ‘Russkie Korni’ store (https://magazintrav.ru, accessed on 13 October 2022). The plants were ground with an ULTRA-TURRAX^®^ Tube Drive homogenizer (IKA; Staufen, Germany) to create a fine, homogeneous powder in order to extract them. This was then followed by mixing 1 g of powder with 30 mL of 90% EtOH. The suspension was heated in a microwave for 5 min total without intensive boiling. The extracted sample was cooled and centrifuged to separate the supernatant from the sediment, and then the supernatant was used. Extracts were kept at +4 °C in containers that were tightly closed.

### 2.2. Autophagy Test on Cell Culture

Human SH-SY5Y cells (the cell line was obtained from Karagyaur Maxim Nikolaevich, Department of Biochemistry and Regenerative Biomedicine, Faculty of Fundamental Medicine, Lomonosov Moscow State University) were cultured in DMEM/F12 medium (Gibco™, New York, NY, USA: 11320033), containing 10% FBS (Gibco™, New York, NY, USA: 16000044), 100 U/mL of penicillin and 100 µg/mL of streptomycin (Gibco™, New York, NY, USA: 15140122), and 1% glutaMAX (Gibco™, New York, NY, USA: 35050061) at 37 °C and 5% CO_2_. Harvesting was performed when reaching 70–80% confluence via trypsinization.

Prior to testing, 50,000 cells per point in 100 µL of culture medium were seeded. The required volume of extract or fraction as a whole was lyophilized by the Labconco FreeZone 2.5 L dryer (Labconco, Kansas City, MO, USA) and then dissolved in 100 µL medium with 1% dimethyl sulfoxide (DMSO, Component-reagent, Moscow, Russia). The mixture was applied to the cells 24 h after plating. Carbonyl cyanide p-trifluoromethoxyphenylhydrazone (FCCP; APExBIO, Hong Kong, China: B5004) was used as a positive control. After 24 h, the cells were harvested and analyzed.

### 2.3. Western Blot

For detection of the proteins, the following antibodies were used: LC3A/B (D3U4C; Cell Signaling, Danvers, MA, USA: 12741) and HRP-conjugated goat anti-rabbit IgG antibodies (Fisher Scientific, Invitrogen, Carlsbad, CA, USA: G21234).

### 2.4. High-Performance Liquid Chromatography (HPLC)

Chemicals: acetonitrile (MeCN) (HPLC grade, Fisher Scientific, Waltham, MA, USA), trifluoroacetic acid (TFA) (HPLC grade, Fisher Scientific, Waltham, MA, USA), and formic acid (FA) (LCMS grade, Fisher Scientific, Waltham, MA, USA).

Analyses were performed on an Agilent 1260 Infinity system (Agilent Technologies, Santa Clara, CA, USA) equipped with a binary pump, dual-loop autosampler, fraction collector, column thermostat, and diode-array detector (DAD) using a Macherey-Nagel C18 5 μm 4.6 × 250 mm column (Macherey-Nagel GmbH & Co. KG, Düren, Germany) at 35 °C. UV detection was achieved at 210, 254, 350, and 500 nm.

*Vaccinium vitis-idaea* extract: mobile phase A—water (with 0.1% TFA); mobile phase B—acetonitrile (MeCN; HPLC grade, Fisher Scientific, Waltham, MA, USA) (with 0.1% TFA). The injection volume was 10 μL. An elution program of 0–4–14–17–20 min and 5–20–30–95–95% B at 1 mL/min was used. The active fraction eluted at ~9.5 min.

*Thymus serpyllum* extract: mobile phase A—water (with 0.1% TFA); mobile phase B—MeCN (with 0.1% TFA). The injection volume was 25 μL. An elution program of 0–3–9–13–16 min and 5–20–20–95–95% B at 1 mL/min was used. The active fraction was eluted at ~10.2 min.

*Saponaria officinalis* extract: mobile phase A—water; mobile phase B—MeCN. The injection volume was 50 μL. An elution program of 0–10–14 min and 5–95–95% B at 1 mL/min was used. The active fraction was eluted at ~6.4 min.

*Veronica officinalis* extract: mobile phase A—water (with 0.1% TFA); mobile phase B—MeCN (with 0.1% TFA). The injection volume was 50 μL. An elution program of 0–3–9–13–16 min and 5–35–35–95–95% B at 1 mL/min was used. The active fractions were eluted at ~7.8 min (chlorogenic acid) and 11.5 min (baicalin).

*Schisandra chinensis* extract: mobile phase A—water (with 0.1% TFA); mobile phase B—MeCN (with 0.1% TFA). The injection volume was 50 μL. An elution program of 0–10–14 min and 70–95–95% B at 1 mL/min was used. The active fractions were eluted at 4.9 min, 9.6 min, and 10.5 min.

*Astragalus dasyanthus* extract: mobile phase A—water (with 0.1% TFA); mobile phase B—MeCN (with 0.1% TFA). The injection volume was 50 μL. An elution program of 0–10–14 min and 5–95–95% B at 1 mL/min was used. The active fraction was eluted at 13.6 min.

### 2.5. High-Resolution Liquid Chromatography–Mass Spectrometry (HR-LCMS)

HR-LCMS analysis. The analysis was performed using an UltiMate 3000 Acclaim RSLC HPLC system (Fisher Scientific, Waltham, MA, USA) with a 120 C18 2.2 um 2.1 × 100 mm column and a gradient of acetonitrile (0.1% FA) in water (0.1% TFA) of 5–95% for 10 min, while connected to a Bruker maXis II 4 G ETD (Bruker, Billerica, MA, USA). Spectra recording mode: ESI (Electrospray Ionization) mode, full scan from 100 to 1500 *m*/*z*, MS/MS with a selection of the three most intense ions. Dissociation type: CID 10–40 eV, collision gas nitrogen. The obtained mass spectra were analyzed using OpenChrom Lablicate Edition (1.5.0) and TOPPView (2.0) software [20]. FBMN was performed following GNPS guidelines [21]. Mass spectra files were processed using the MzMine package (2025, 4.8.5) under the following conditions: instrument—HPLC-QTOF-DDA (ion mode: positive), added to the Isotopic peaks’ finder module. Processed files were uploaded to the GNPS FBMN workflow (for schisandrin, the results are accessible at https://gnps.ucsd.edu/ProteoSAFe/status.jsp?task=a60d948607334a309093db4ed733f8d7, accessed on 15 February 2025). The FBMN results were visualized using Cytoscape (2025, 3.10.4) for network analysis. All graphical representations were further refined and finalized in Adobe Illustrator (2025, version 29.6.0.207).

## 3. Results

### 3.1. Primary Screening of Plant Extracts for Their Ability to Induce Autophagy

In this study, we aimed to establish and validate a screening method for identifying potential sources of autophagy inducers. To prepare ethanolic extracts, 1 g of dried plant material was boiled in 30 mL of 90% ethanol. Since the study of autophagy inducers in medicinal plants is inextricably linked with the use of the whole extracts and individual components in traditional and modern medicine. Ethanol extraction was selected as a rapid and relatively safe method for obtaining highly concentrated solutions of biologically active compounds [22,23,24].

The required volumes of ethanolic extract were evaporated to dryness and applied to SH-SY5Y cells in culture medium containing 1% DMSO to improve the solubility of bioactive compounds. Cells were incubated with the extracts for 24 h, after which autophagy levels were analyzed.

LC3 (microtubule-associated protein 1A/1B light chain 3) was selected as a marker of autophagy. Upon autophagy activation, the cytosolic form LC3 I conjugates with phosphatidylethanolamine to form LC3 II, which is incorporated into the membrane of the forming autophagosome and degraded together with the autophagic substrate [25]. An increase in LC3 II levels serves as a marker of autophagosome accumulation and is commonly used as an indicator of autophagy activation [26]. We employed Western blot analysis with antibodies against LC3 I/II (recognizing both LC3A and LC3B) as a simple and accessible method to evaluate the activity of plant extracts or their fractions.

In this work, we focused on identifying plants containing relatively high levels of autophagy inducers that could potentially serve as sources for industrial-scale isolation. To estimate the concentration range of total extracts capable of activating autophagy, *Veronica officinalis* was selected as a model. Plants of the genus *Veronica* contain numerous bioactive metabolites [27], including autophagy inducers [28]. It has also been reported that a phenolic-rich fraction of the ethanolic extract of *Veronica ciliata* modulates autophagy via activation of the AMPK/p62/Nrf2 pathway [29].

**Figure 1:**
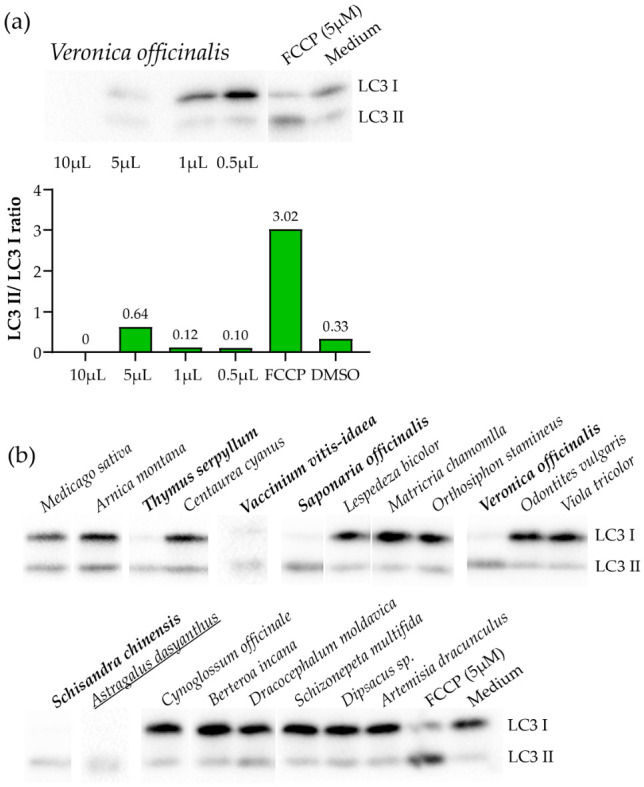
Primary screening of the ability of ethanolic extracts from various plants to induce autophagy, assessed by Western blot analysis using antibodies against LC3 I/II. (**a**) Determination of the amount of ethanolic extract from *Veronica officinalis* required to induce autophagy in SH-SY5Y cells (full image shown in Figure S1a). Densitometric analysis of the LC3 II/LC3 I ratio is shown under the staining result; here and further in the text increase in the LC3 II/LC3 I ratio is considered a marker of autophagy. (**b**) Analysis of the ability of ethanolic extracts from various plants to induce autophagy (full image and densitometric analysis are shown in Figure S1b). Plants previously reported in the literature to induce autophagy (Table 1) are shown in bold. The newly identified autophagy inducer, *Astragalus dasyanthus*, is underlined.

Volumes of ethanolic extract corresponding to 10, 5, 1, and 0.5 µL were evaporated and applied to SH-SY5Y cells. Based on Western blot analysis of autophagy induction by these extract amounts (Figure 1a), we selected 5 µL per sample as the standard volume for further screening. While this choice inherently limited the sensitivity of the screening to extracts containing autophagy inducers at levels not lower than those in *Veronica officinalis*, the baseline conditions can be adjusted depending on the specific research objectives.

To confirm the ability of the method to detect major autophagy inducers, five plants (including *Veronica officinalis*) known to be sources of autophagy-inducing compounds were selected (Table 1).

In addition, fifteen plant species with no previously reported direct association with autophagy were analyzed (Figure 1b). Ethanolic extracts (5 µL per sample) were applied to SH-SY5Y cells. In cases where cell death was observed—without excluding excessive autophagy as a possible cause—the applied volume was reduced (final working volumes are provided in Table S1).

Ultimately, all six control plants induced autophagy (Figure 1b). Notably, one extract from the test group (*Astragalus dasyanthus*) demonstrated autophagy-inducing activity comparable to that of the control plants (Figure 1b).

### 3.2. Identification of the Active Compound in Saponaria officinalis

The ethanolic extract of *Saponaria officinalis* was fractionated by HPLC (see Section 2 for detailed chromatography procedure). UV detection of chemical compounds in the fractions was performed at 210, 254, 350, and 500 nm.

**Figure 2:**
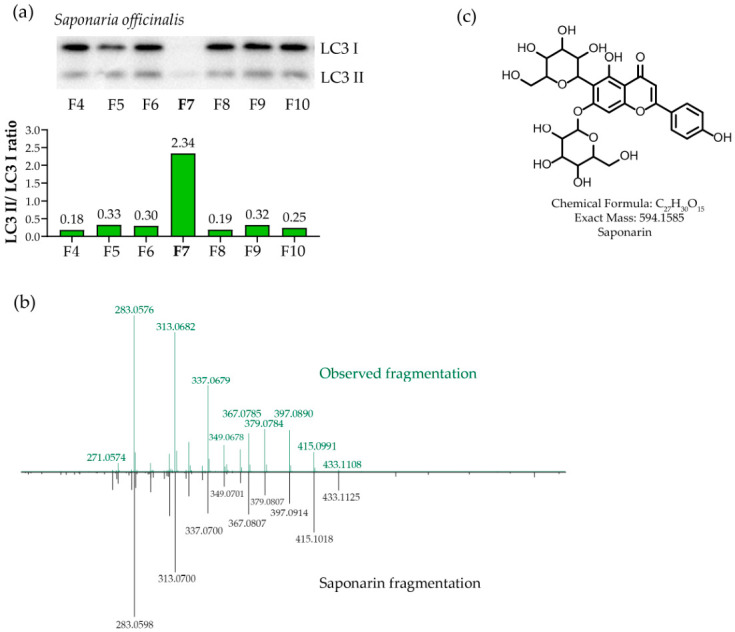
Identification of the active compound in *Saponaria officinalis*. (**a**) Western blot analysis of the ability of HPLC fractions of the ethanolic extract of *Saponaria officinalis* to induce autophagy (LC3 I/II staining: the panel shows the fragment containing the active fraction; the full image is presented in Figure S2a; densitometric analysis of the LC3 II/LC3 I ratio is shown under the staining result; the fraction sent for further analysis is marked in bold). (**b**) MS/MS comparison of the observed ion in positive-ion mode (top graph, *m*/*z* 595.156) with the deposited GNPS reference spectrum for saponarin [M + H]^+^ (bottom graph, GNPS LIBRARY: accession: CCMSLIB00010125140, *m*/*z* 595.1660). (**c**) Chemical structure of saponarin.

The collected fractions were completely dried and analyzed for their ability to induce autophagy in SH-SY5Y cells (Figure 2a and Figure S2a). Fraction 7 (F7) demonstrated the highest activity. F7 of *Saponaria officinalis* extract was dominated by a single major active component with UV-Vis absorption maxima at 270 and 337 nm, suggesting a flavone chromophore. HR-LC MS revealed adducts consistent with the chemical formula C_27_H_30_O_15_ ([M + H]^+^ calculated 595.1663, found 595.1627, Δ = 0.6 ppm; [M + H]^-^ calculated 593.1505, found 593.1522, Δ = 3 ppm). MS/MS fragmentation in both ionization modes (Figure 2b and Figure S3) matched the known glycosylated flavone saponarin (Figure 2c).

### 3.3. Identification of the Autophagy Inducer in Veronica officinalis

**Figure 3:**
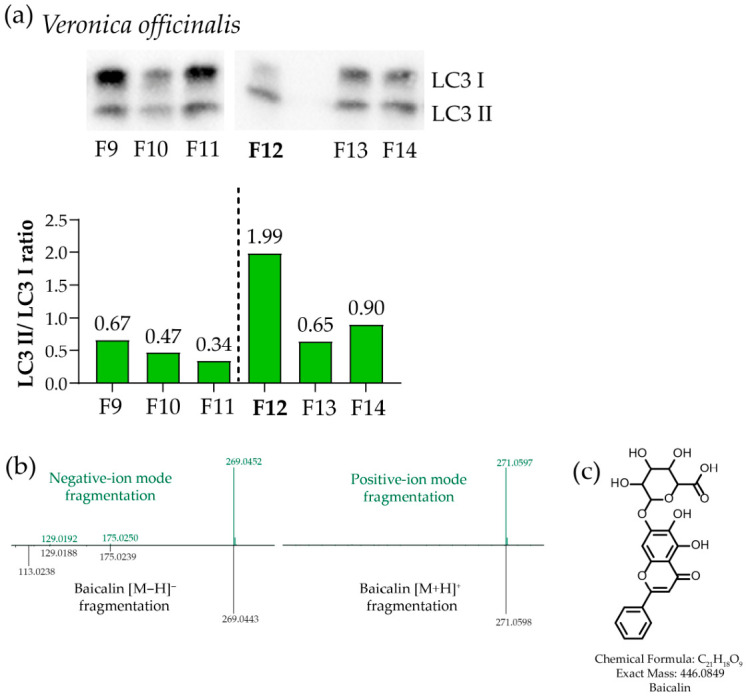
Identification of the active compounds in *Veronica officinalis*. (**a**) Western blot analysis of the ability of HPLC fractions of the ethanolic extract of *Veronica officinalis* to induce autophagy (LC3 I/II staining: the panel shows the fragment containing the active fraction; the full image is presented in Figure S2b, and densitometric analysis of the LC3 II/LC3 I ratio is shown under the staining result; the fraction sent for further analysis is marked in bold). (**b**) MS/MS comparison of the observed ions in positive-ion mode (*m*/*z* 447.091) and negative-ion mode (*m*/*z* 445.076) with the deposited GNPS reference spectra for baicalin [M + H]^+^ and [M − H]^−^ (GNPS-LIBRARY: accession: CCMSLIB00013029693, *m*/*z* 447.092; GNPS-LIBRARY: accession: CCMSLIB00012855511, *m*/*z* 445.078, respectively). (**c**) Chemical structure of baicalin.

Following analogous HPLC fractionation of *Veronica officinalis* extract, fraction 12 (F12) showed the highest activity (Figure 3a and Figure S2b). An active fraction yielded ions consistent with C_21_H_18_O_11_: [M + H]^+^ at 447.0912 (calculated 447.0927, Δ = 3 ppm) and [M − H]^−^ at 445.0767 (calculated 445.0771, Δ = 0.9 ppm). The MS/MS fragmentation patterns in both modes featured a dominant fragment ion (Figure S4a,b), supporting assignment to the glycosylated oxyflavone baicalin (Figure 3b,c).

### 3.4. Identification of Autophagy Inducers in Vaccinium vitis-idaea, Thymus serpyllum, and Schisandra chinensis

**Figure 4:**
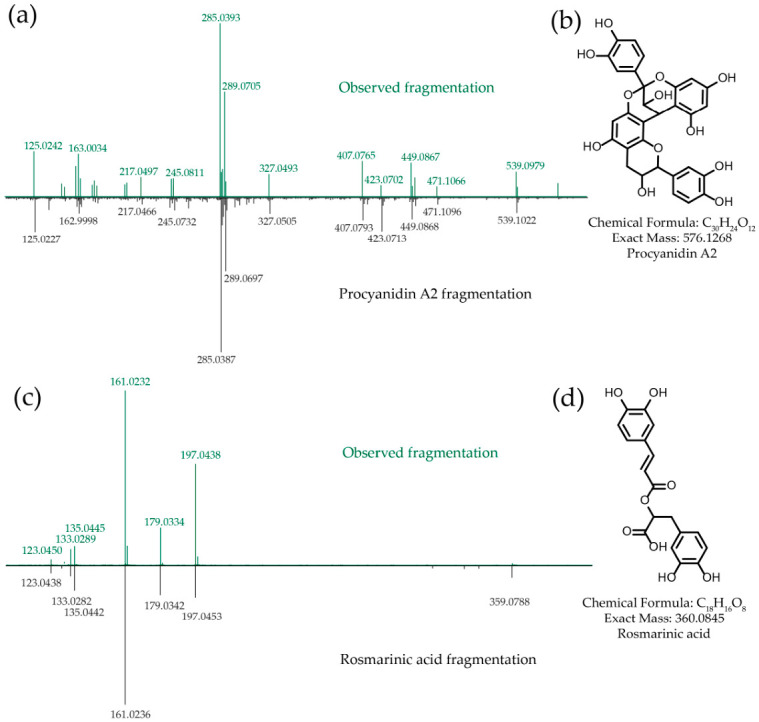
Identification of autophagy inducers in *Vaccinium vitis-idaea*, *Thymus serpyllum*. (**a**) MS/MS comparison of the observed ion in negative-ion mode (top graph, *m*/*z* 575.1182) with the deposited GNPS reference spectrum for procyanidin A2 [M − H]^−^ (bottom graph, GNPS-LIBRARY; accession: CCMSLIB00012176091, *m*/*z* 575.1210). (**b**) Chemical structure of procyanidin A2. (**c**) MS/MS comparison of the observed ion in negative-ion mode (top graph, *m*/*z* 359.0742) with the deposited GNPS reference spectrum for rosmarinic acid [M − H]^−^ (bottom graph, GNPS-LIBRARY; accession: CCMSLIB00005778380, *m*/*z* 359.0770). (**d**) Chemical structure of rosmarinic acid.

The principal active compounds were also identified for the remaining control plants. The active fraction of *Vaccinium vitis-idaea* ethanolic extract (Figure S2c; F11) was analyzed by HR-LCMS. In negative-ion mode, an ion at *m*/*z* 575.1181 was detected and assigned to the [M + H]^-^ adduct of procyanidin A2 (Figure 4b; C_30_H_24_O_12_, calculated [M − H]^−^ 575.1190, Δ = 1.4 ppm). The composition was corroborated by the corresponding [M − H]^+^ adduct at *m*/*z* 577.1327 (calculated [M + H]^+^ 577.1346, Δ = 3.3 ppm). MS/MS spectra were acquired in both ionization modes (Figure 4a and Figure S5). Fragmentation of the protonated species produced a dominant fragment at *m*/*z* 287.0546, consistent with a monomeric unit (Figure S5a). The [M − H]^−^ fragmentation pattern was in agreement with prior reports (Figure 4a and Figure S5b) and displayed fragment ions at *m*/*z* 285 and 289, characteristic of an A-type procyanidin dimer [38].

Activity-guided fractionation of *Thymus serpyllum* extract afforded a single active compound (Figure S2d; F12) with prominent UV-Vis maxima at 331 nm and 288 nm. HR-LCMS in negative-ion mode showed a [M − H]^−^ ion at *m*/*z* 359.0742, while positive-mode adducts were of low intensity. The measured adduct mass is consistent with the chemical formula C_18_H_16_O_8_ (calculated [M − H]^−^ 359.0767, Δ = 7 ppm). The fragmentation pattern (Figure S6) was identical to that of deprotonated rosmarinic acid (Figure 4c,d).

**Figure 5:**
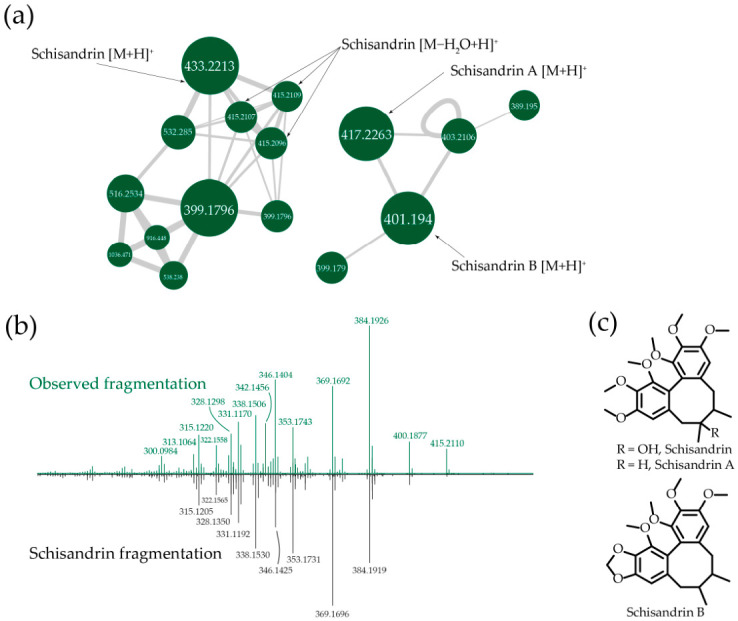
Identification of autophagy inducers in *Schisandra chinensis*. (**a**) Fragment of a feature-based molecular network obtained from HR-LCMS spectra of the isolated fractions. Each node in the molecular network represents a feature derived from an XIC (extracted ion chromatogram). The size of the nodes corresponds to the combined abundance of the respective feature across all samples. Edges between nodes indicate tandem mass-spectra correlation, with edge width proportional to the cosine score. Each node is labeled with the *m*/*z* of the corresponding feature. (**b**) MS/MS comparison of the observed ion in positive-ion mode (top graph, *m*/*z* 433.221) with the deposited GNPS reference spectrum for schisandrin [M + H]^+^ (bottom graph, GNPS-LIBRARY; accession: CCMSLIB00005744455, *m*/*z* 433.2220). (**c**) Chemical structures of schisandrins.

Fractionation of *Schisandra chinensis* ethanolic extract yielded several hydrophobic active fractions, of which some of the most active were analyzed (Figure S2e; F4, F15, F17). These fractions produced signals only in positive-ion mode. Given their similar UV-Vis absorption maxima, we hypothesized a set of structurally related metabolites and analyzed LC HRMS data using GNPS feature-based molecular networking (Figure 5a). The network revealed a cluster of closely related nodes with [M + H]^+^ ions at *m*/*z* 433.2213, 417.2263, and 401.1951, and similar MS/MS patterns (Figure S7). The one component was identified as schichandrin (Figure 5b,c; C_24_H_32_O_7_, calculated [M + H]^+^ 433.2226, Δ = 3 ppm). The remaining nodes are consistent with schisandrin A (C_24_H_32_O_6_, calculated [M + H]^+^ 417.2277, Δ = 3 ppm) and schisandrin B (C_23_H_28_O_6_, calculated [M + H]^+^ 401.1964, Δ = 3 ppm), based on exact mass and shared fragmentation features.

### 3.5. Identification of Autophagy Inducers in Astragalus dasyanthus

**Figure 6:**
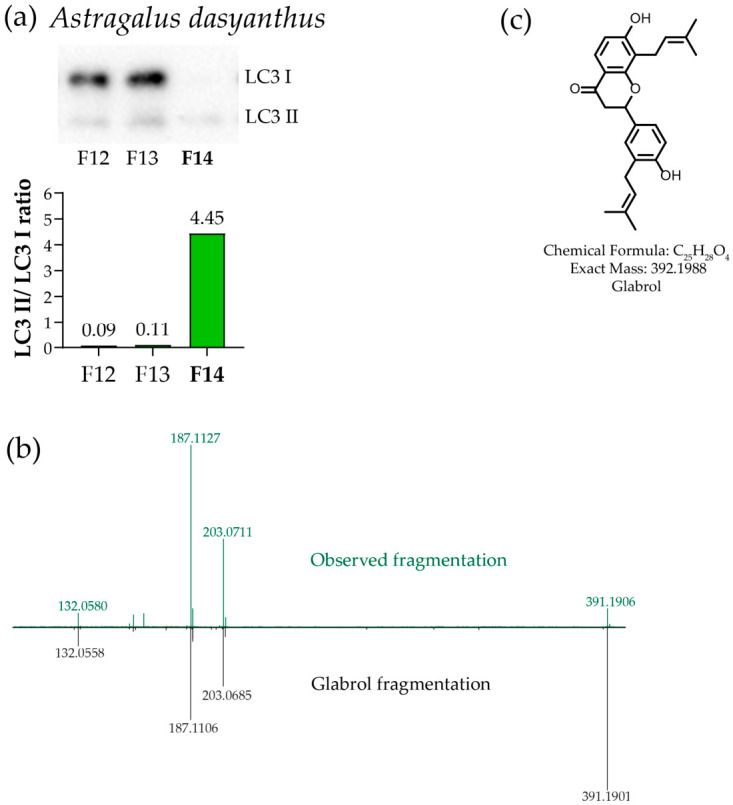
Identification of the active compounds in *Astragalus dasyanthus*. (**a**) Western blot analysis of the ability of HPLC fractions of the ethanolic extract of *Astragalus dasyanthus* to induce autophagy (LC3 I/II staining: the panel shows the fragment containing the active fraction; the full image is presented in Figure S2f; the fraction sent for further analysis is marked in bold). (**b**) MS/MS comparison of the observed ion in negative-ion mode (*m*/*z* 391.1910) with the reference spectrum for glabrol [M − H]^−^ (GNPS-LIBRARY; accession: CCMSLIB00005749603, *m*/*z* 393.19). (**c**) Chemical structure of glabrol.

The literature reports that a mixture of aqueous extracts from the related plants *Astragalus membranaceus* and *Cornus officinalis* induces autophagy in mice via the PI3K–AKT pathway; however, the individual contribution of each plant was not examined [39]. Astragalus polysaccharide, a component of *Astragalus membranaceus*, has also been shown to enhance podocyte autophagy via the SIRT1/FOXO3a/BNIP3 pathway in a diabetes model [40]. Thus, it could have been hypothesized that *Astragalus dasyanthus* extract would also positively affect autophagy; however, no direct evidence was found in the literature. As demonstrated in the primary screening, *Astragalus dasyanthus* extract increased the LC3 II/LC3 I ratio in SH-SY5Y cells. Following HPLC fractionation of the ethanolic extract (see Section 2 for details), activity was detected in the most hydrophobic fraction (Figure 6a and Figure S2f F14). LC–HRMS analysis of the hydrophobic fraction revealed a compound with the molecular formula C_25_H_28_O_4_ ([M + H]^+^ calculated 393.2066, found 393.2045, Δ = 5 ppm; [M − H]^−^ calculated 391.1909, found 391.1910, Δ = 0.3 ppm). MS/MS fragmentation (Figure S8) suggested that the isolated compound could be identified as the diprenylflavanone glabrol (Figure 6c) based on characteristic fragment ions observed in negative-ion mode (Figure 6b and Figure S8b).

## 4. Discussion

Maintaining an optimal level of autophagy is essential both for the normal functioning of individual cells and for preventing pathological conditions in the organism. Autophagy is regulated by a complex network of proteins, and disruption of this regulation at different stages leads to the development of various diseases, such as diabetes and Alzheimer’s disease [41,42]. Consequently, the search for and study of autophagy modulators represents a promising area of research. Natural substrates often serve as sources of medicinal compounds, including various autophagy inducers [43]. The vast diversity of the plant kingdom provides substantial material for investigation. In this article, we describe an HR-LCMS/MS-based technology for identifying the principal compound in plant extracts capable of activating autophagy. The described method, like many similar ones, is based on the analysis of the LC3 II/I ratio [44,45], but does not require the creation of additional cell lines, as the analysis of the ratio is carried out using the Western blot method.

HR-LCMS/MS is a powerful technique for determining the composition of a multi-component plant extract [46,47]. However, often a complete determination of all the main components of the extract is first carried out, and then those responsible for the intended function are found [48,49]. In this work, we propose the inclusion of an additional stage of testing HPLC fractions using the Western blot method and targeted determination of only the main substance responsible for the induction of autophagy. This addition allows for more efficient, high-throughput searches for natural autophagy inducers while simultaneously reducing the number of substances requiring analysis and increasing the specificity of bioactive substance determination. Despite inherent limitations of LC–MS/MS-based metabolite identification, particularly the frequent inability to unambiguously assign full structures (e.g., stereochemistry and regioisomerism) without authentic standards, this approach can be effectively used for rapid dereplication and scaffold-level annotation, enabling primary prioritization of bioactive natural products [50,51,52].

To prove the effectiveness of the described method, we identified the major bioactive substances in plants associated with autophagy activation, namely *Saponaria officinalis, Veronica officinalis, Vaccinium vitis-idaea*, *Thymus serpyllum*, and *Schisandra chinensis*.

It is known that the main bioactive compounds in the composition of plants of the genus *Saponaria* are saponins, some of which are also capable of activating autophagy [30,31,32]. After analyzing the main autophagy-inducing fraction of the alcoholic extract of *Saponaria officinalis*, we found saponarin there. Previous studies have demonstrated the presence of significant amounts of saponarin in extracts of *Saponaria officinalis* [53], while the saponarin content in plant extracts is known to determine their ability to activate autophagy via the AMPK signaling pathway [54], demonstrating the consistency of our findings.

The ability of certain chromatographic fractions of *Veronica* plant extract to activate autophagy via the AMPK/p62/Nrf2 pathway is known from the literature [29]. We detected baicalin in the active HPLC fraction of the ethanolic extract of *Veronica officinalis*. Baicalin modulates the AMPK signaling cascade, enhancing autophagy [55], and has been detected in plants of the genus *Veronica* [56,57].

Powder from the plant *Vaccinium vitis-idaea* has been shown to prevent nonalcoholic fatty liver disease-related expression changes in the livers of mice on a high-fat diet [34], implying the ability of this plant to activate autophagy. According to our data, one of the main active substances in *Vaccinium vitis-idaea* extract is procyanidin A2, which induced autophagy and improved insulin sensitivity in a mouse model of diabetes [58]. Taking into account that this compound is present in significant amounts in plants of the genus *Vaccinium* [59], it is possible to assume that procyanidin A2 is responsible for the hepatoprotective effect of *Vaccinium vitis-idaea* in mice on a high-fat diet.

As for *Thymus serpyllum* and *Schisandra chinensis*, these plants contain autophagy inducers that are specific to them, and these specific compounds have been identified. In *Thymus serpyllum,* we detected rosmarinic acid, a typical *Thymus* sp. metabolite [60,61], known to activate autophagy [62,63]. Schisandrin A, a typical metabolite of *Schisandra chinensis* [64], was previously shown to induce autophagy in SH-SY5Y cells by directly targeting the Ykt6 protein [65].

As described above, our observations are fully consistent with those in the literature, demonstrating the accuracy of the described method. However, although the composition of plant extracts is commonly known, a direct experimental link between the induction of autophagy by the extract and its main bioactive component has not always been established. For example, we demonstrated that saponarin is the main compound responsible for the autophagic effect of *Saponaria officinalis*. It is also important to remember that the autophagy inducer we have described may not be the only one in the current plant extract, but only the one contained in the highest active concentration, which is both a limitation and an advantage of the method, allowing us to simultaneously isolate the autophagy activator and show that it is contained in a relatively high active concentration.

To show the effectiveness of the described method, we analyzed fifteen plants for which the ability to induce autophagy had not previously been demonstrated. We found that the alcoholic extract of *Astragalus dasyanthus*, when applied to SH-SY5Y cells, caused an increase in the amount of LC3 II compared to LC3 I. After analyzing the main active fraction, we discovered the substance glabrol in its composition. Glabrol, isolated from the roots of *Glycyrrhiza inflata*, is a known inhibitor of PTP1B [66], which makes it a potential inducer of autophagy in various models [67]. It has also been shown that glabrol is the principal metabolite responsible for autophagy induction in mice treated with Taohong Siwu Decoction [68]. However, the presence of glabrol among the metabolites of *Astragalus dasyanthus* has been identified here for the first time [69].

Considering further possibilities for the development of the screening method for plant inducers of autophagy based on the combination of HR-LCMS/MS and Western blot, we can propose the use of additional autophagy markers. As an increase in the LC3 II/I ratio indicates an accumulation of autophagosomes in the cytoplasm, it provides limited information about the mechanism of autophagy activation [25]. This analysis can be complemented by Western blot analysis of any other autophagy markers; for example, antibodies to PINK1 and Parkin proteins can be used to search for specific substances activating the PINK1/Parkin-mediated mitophagy [70]. At the same time, the use of different solvents to obtain extracts can significantly expand the range of substances studied. Methanol can serve as an alternative to ethanol for the extraction of polar compounds, and solvents such as hexane can be used for the extraction of nonpolar compounds [22].

In summary, the HR-LCMS/MS-based method for identifying key autophagy-activating plant components described in this article can be successfully used to find the main autophagy-inducing substance in plants with a known composition, as well as to search for new producers of known bioactive substances, as shown here for the detection of glabrol in *Astragalus dasyanthus*. However, the most promising direction of further research is the high-throughput screening for new, previously undiscovered autophagy activators followed by the isolation of the new compounds, confirmation of their structures using pure standards, and study of their working concentrations and mechanisms of action for future medicinal use.

## 5. Conclusions

In this paper, we proposed an HR-LCMS/MS-based method for identifying the primary autophagy inducer in a plant extract. We demonstrated both the accuracy of this method by correlating the results obtained for five autophagy-activating plants with the literature data and its effectiveness by demonstrating the presence of significant amounts of glabrol in *Astragalus dasyanthus* extract. In its current configuration, the method is optimized for the rapid screening of a large set of plant substrates, both to identify new producers of known autophagy inducers and to search for new substances that, after additional research, can be successfully used in modern medicine to treat and alleviate a wide range of diseases.

## Figures and Tables

**Table 1:** Plants known to induce autophagy (used in the study as method control).

Plant	Form	Known Link to Autophagy
*Saponaria officinalis*	Individual substances	A well-known source of saponins (some induce autophagy [30,31,32])
*Veronica officinalis*	Ethyl acetate extract chromatography fractions	Activate the AMPK/p62/Nrf2 pathway in mouse liver [29,33]
*Vaccinium vitis-idaea*	Powder	Prevents liver changes in mice on a high-fat diet [34]
*Thymus serpyllum*	Chloroform fraction	Induce autophagy in human NCI-H929 cells [35]
*Schisandra chinensis*	Individual substances	Depends on the substance [36,37]

## Data Availability

The original contributions presented in this study are included in the article/Supplementary Material. Further inquiries can be directed to the corresponding authors.

## Supplementary Materials

The following supporting information can be downloaded at https://www.mdpi.com/article/10.3390/metabo16050311/s1: Figure S1: Analysis of the ability of ethanolic extracts of various plants to induce autophagy in SH-SY5Y cells. Figure S2: Analysis of the ability HPLC fractions of plant ethanolic extracts to induce autophagy in SH-SY5Y cells. Figure S3: MS/MS fragmentation spectra for the compound isolated from *Saponaria officinalis*. Figure S4: MS/MS fragmentation spectra for baicalin isolated from *Veronica officinalis*. Figure S5: MS/MS fragmentation spectra for the compound isolated from *Vaccinium vitis-idaea* ethanolic extract. Figure S6: Fragmentation spectrum of the [M − H]^−^ parent ion at m/z 359.0742 for compound, isolated from *Thymus serpyllum*. Figure S7: MS/MS fragmentation spectra for the compounds isolated from *Schisandra chinensis*. Figure S8: MS/MS fragmentation spectra for the compound isolated from *Astragalus dasyanthus*. Table S1: A complete list of plants used in the work, with working concentrations, if applicable.

## Abbreviations

The following abbreviations are used in this manuscript:

TFA	Trifluoroacetic Acid
FA	Formic Acid
