# Supplementary material for: HR-LCMS/MS-Based Dereplication of Plant-Derived Autophagy Inducers Revealed Astragalus dasyanthus as a New Glabrol Producer
Source: Metabolites. 2026 May 1;16(5):311. doi: 10.3390/metabo16050311 (PMC13208147; doi:10.3390/metabo16050311)
Supplement: Supplementary file 1 [file metabolites-16-00311-s001.zip › Rev_Bolikhova_Supplementary materials.pdf]

Supplementary Materials for the Article: HR-LCMS/MS Based Dereplication of Plant-Derived Autophagy Inducers Revealed *Astragalus Dasyanthus* as a New Glabrol Producer

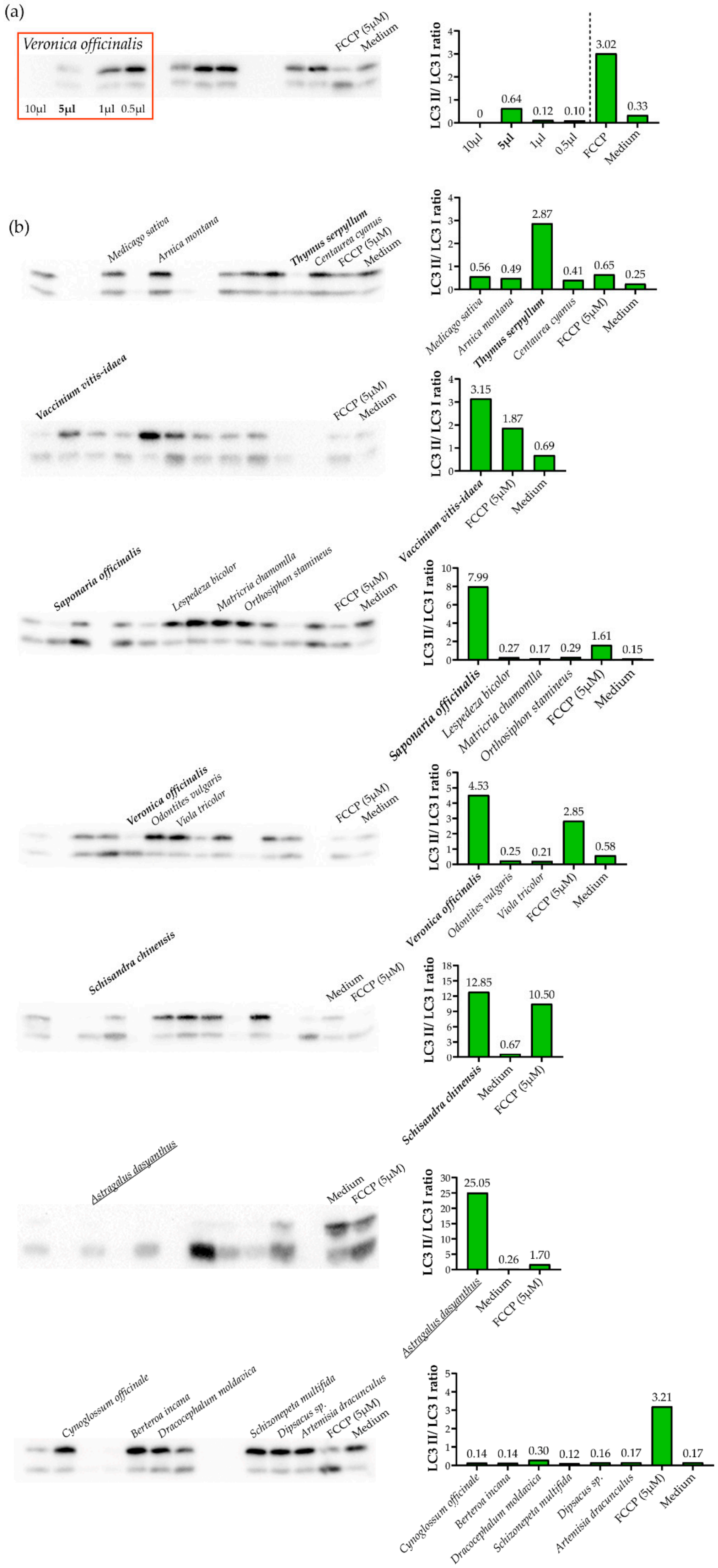
(a)
Veronica officinalis
10µl
5µl
1µl
0.5µl
FCCP (5µM)
Medium
LC3 II/ LC3 I ratio
0
0.64
0.12
0.10
3.02
0.33
FCCP
(b)
Medicago sativa
Arnica montana
Thymus serpyllum
Centaurea cyanus
0.56
0.49
2.87
0.41
0.65
0.25
Vaccinium vitis-idaea
3.15
1.87
0.69
Saponaria officinalis
Lespedeza bicolor
Matricria chamomilla
Orthosiphon stamineus
7.99
0.27
0.17
0.29
1.61
0.15
Veronica officinalis
Odontites vulgaris
Viola tricolor
4.53
0.25
0.21
2.85
0.58
Schisandra chinensis
12.85
0.67
10.50
Astragalus dasyanthus
25.05
0.26
1.70
Cynoglossum officinale
Berteroa incana
Dracocephalum moldavica
Schizonepeta multifida
Dipsacus sp.
Artemisia dracunculus
0.14
0.14
0.30
0.12
0.16
0.17
3.21
0.17

Figure S1. Analysis of the ability of ethanolic extracts of various plants to induce autophagy in SH-SY5Y cells; analysis was performed by western blot with LC3-I/II staining. FCCP at 5 μM was applied to cells as a positive control, and medium with 1% DMSO was used as a negative control. For each image, the staining result is shown on the left, and the densitometric analysis of the LC3 II/LC3 I ratio is shown on the right. (a) Optimization of the volume of *Veronica officinalis* ethanolic extract capable of inducing autophagy in SH-SY5Y cells. (b) Original blots from the primary screening of ethanolic plant extracts for autophagy-inducing activity. Samples corresponding to the extracts mentioned in the text are indicated. Plants previously reported in the literature to induce autophagy (Table 1) are shown in bold. The newly identified autophagy inducer, *Astragalus dasyanthus*, is underlined.

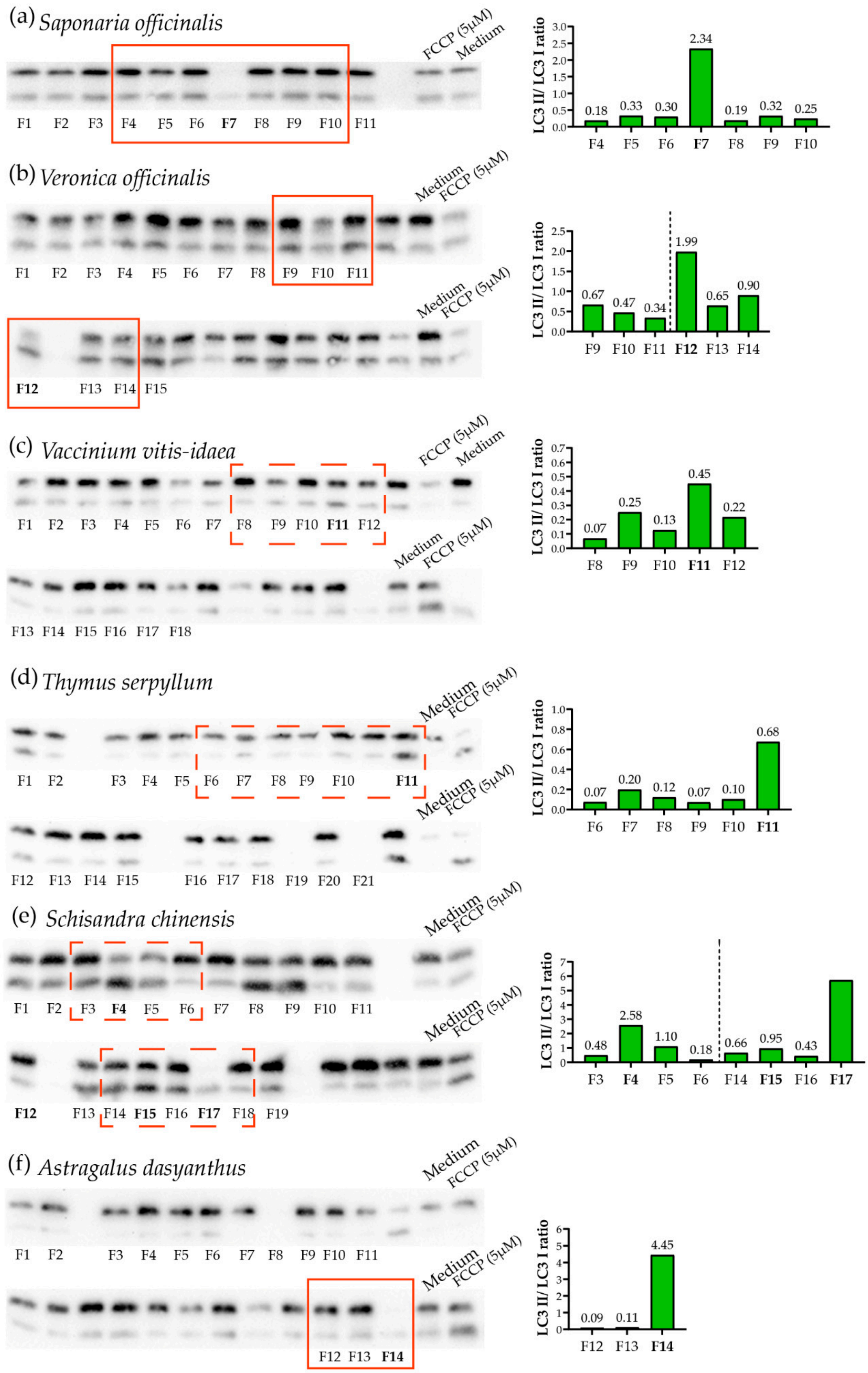

Figure S2. Analysis of the ability HPLC fractions of plant ethanolic extracts to induce autophagy in SH-SY5Y cells: *Saponaria officinalis* (a), *Veronica officinalis* (b), *Vaccinium*

vitis-idaea (c), *Thymus serpyllum* (d), *Schisandra chinensis* (e), and *Astragalus dasyanthus* (f). Fragments containing autophagy-activating fractions chosen for further analysis are marked with a red border: solid (shown in the main text) and dotted (not shown in the main text). Densitometric analysis of the LC3 II/LC3 I ratio for areas of interest are shown on the right. FCCP applied to cells at 5 μM was used as a positive control, and medium with 1% DMSO as a negative control.

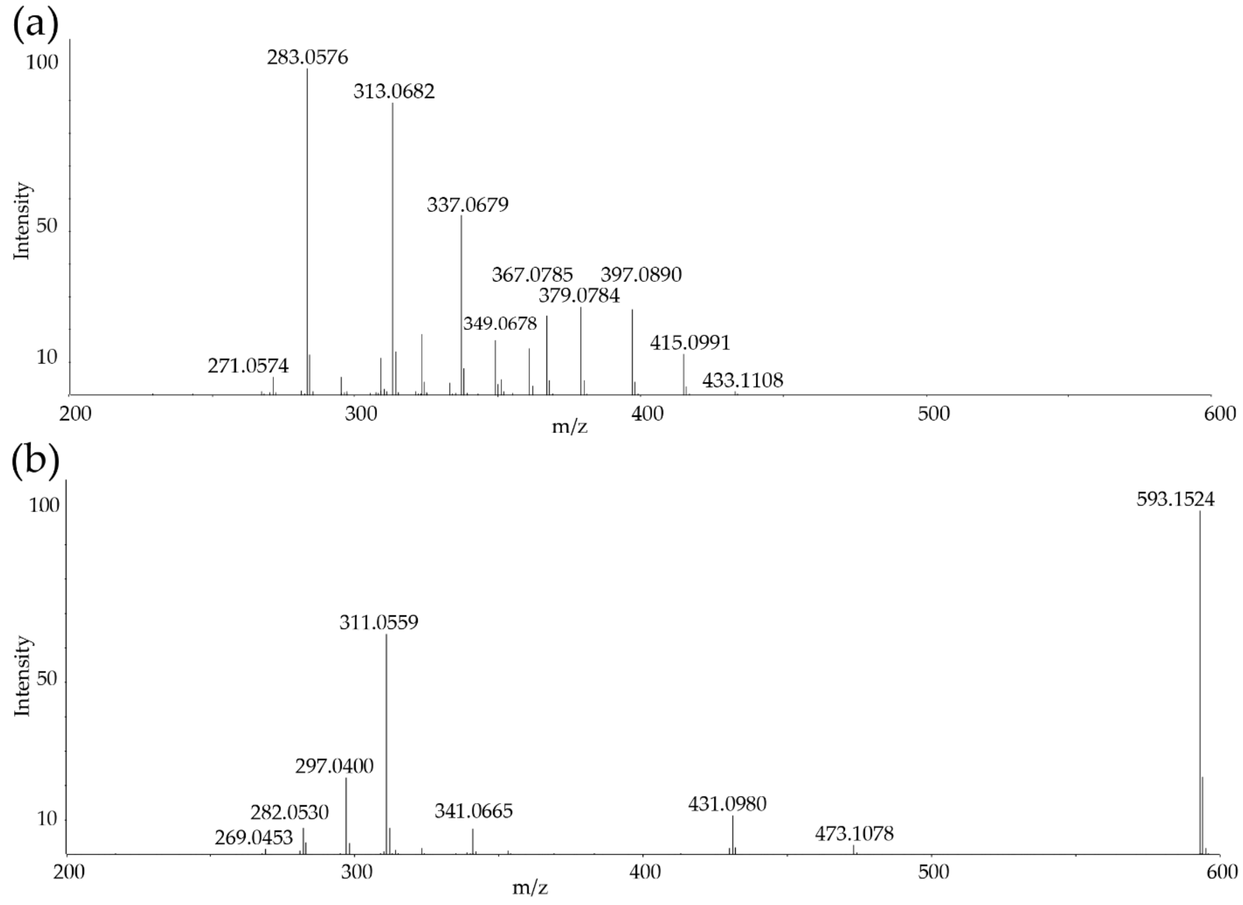

Figure S3. MS/MS fragmentation spectra for the compound isolated from *Saponaria officinalis*. (a) Positive-ion mode, CID spectrum of the precursor ion at *m/z* 595.156. (b) Negative-ion mode, CID spectrum of the precursor ion at *m/z* 593.153.

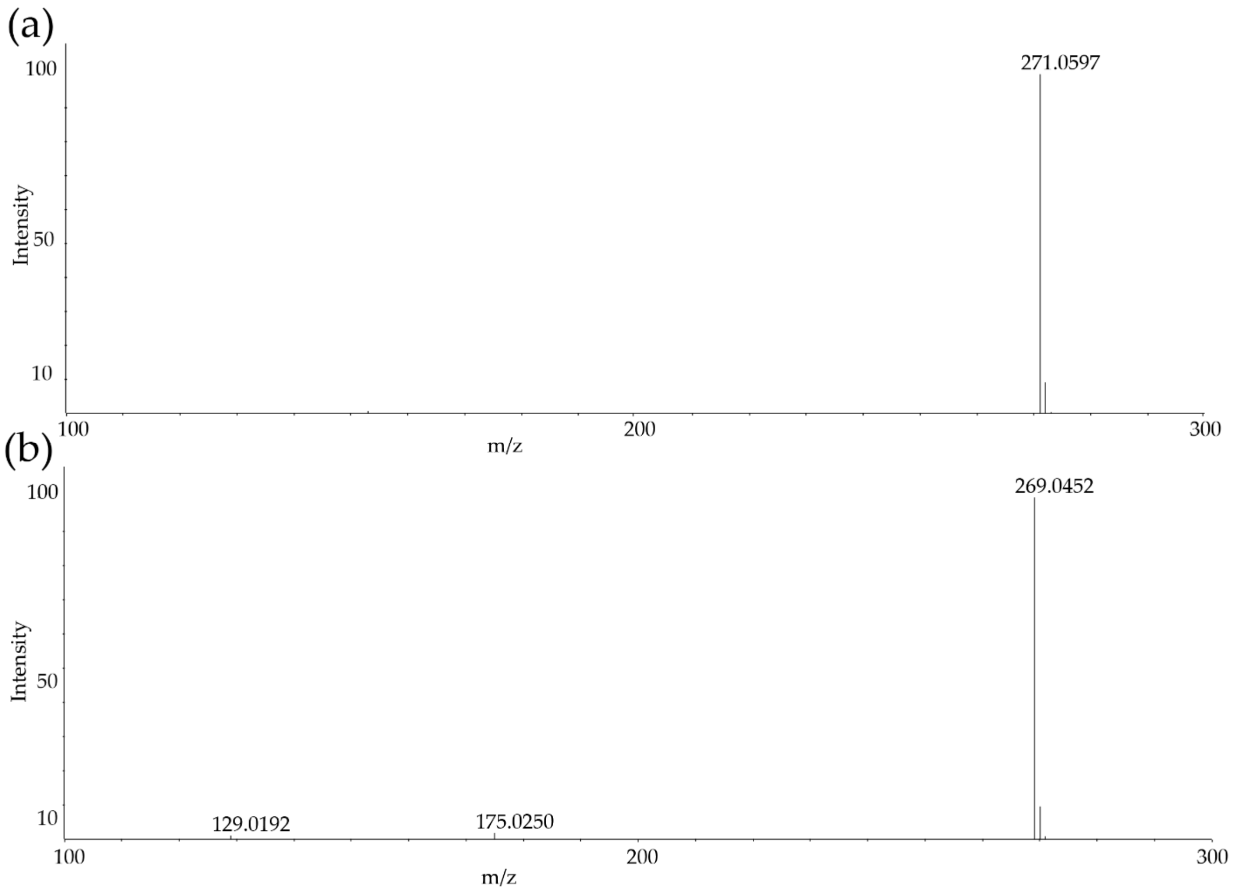

Figure S4. MS/MS fragmentation spectra for baicalin isolated from *Veronica officinalis*. (b) F12, positive-ion mode, CID spectrum of the precursor ion at *m/z 447.091*. (c) F12 negative-ion mode, CID spectrum of the precursor ion at *m/z* 445.076.

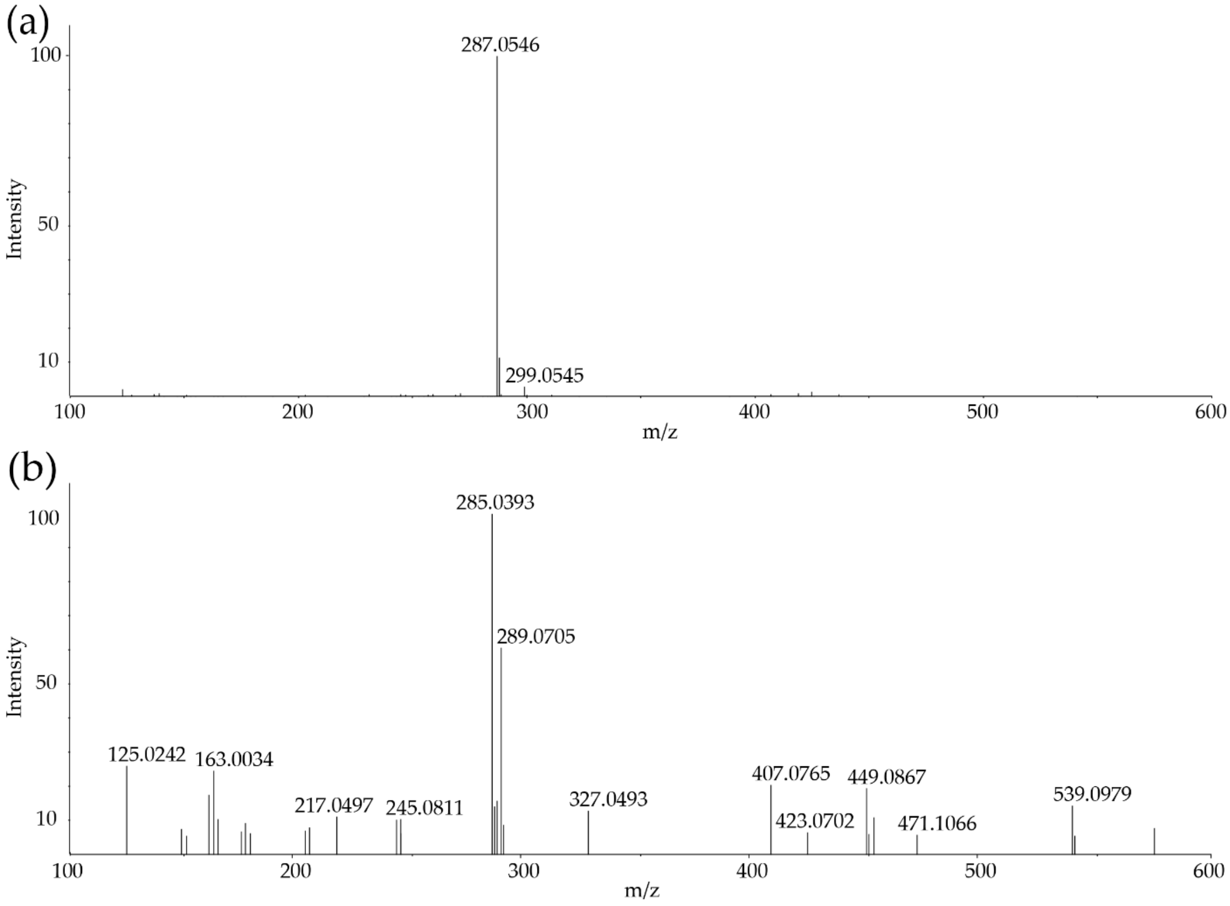

Figure S5. MS/MS fragmentation spectra for the compound isolated from *Vaccinium vitis-idaea* ethanolic extract. (a) Positive-ion mode, CID spectrum of the precursor ion at *m/z* 577.1327. (b) Negative-ion mode, CID spectrum of the precursor ion at *m/z* 575.1182.

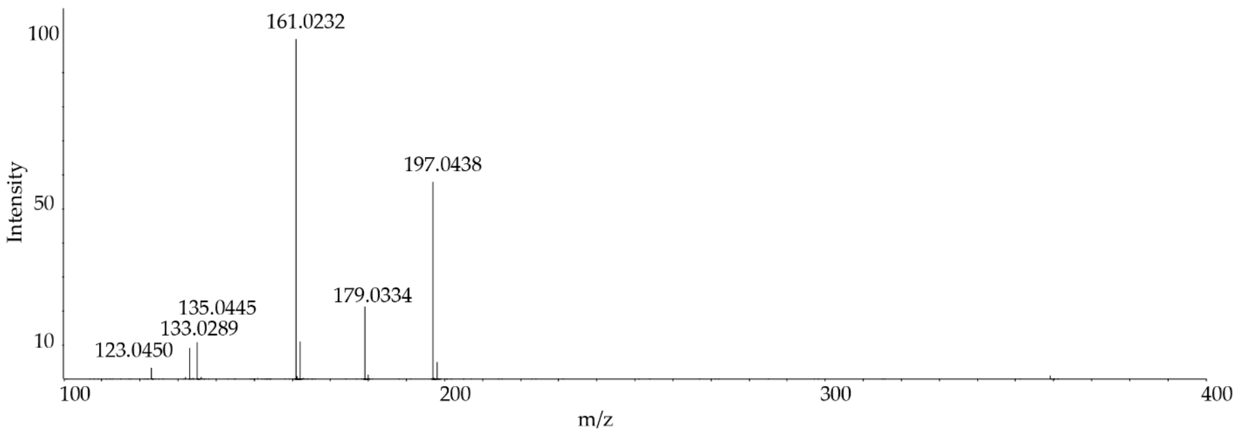

Figure S6. Fragmentation spectrum of the [M-H]- parent ion at m/z 359.0742 for compound, isolated from *Thymus serpyllum*.

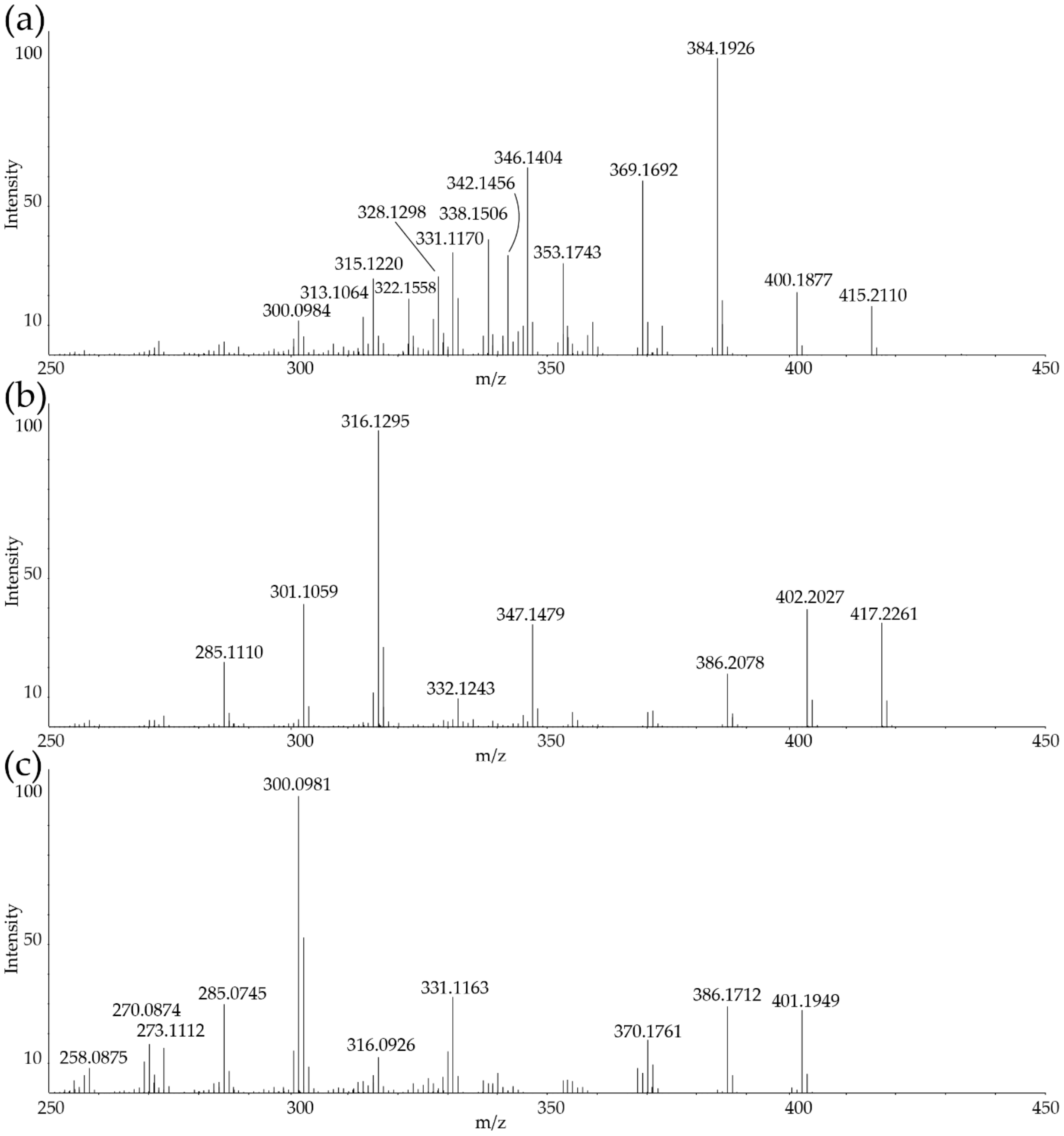

Figure S7. MS/MS fragmentation spectra for the compounds isolated from *Schisandra chinensis*. (a) Positive-ion mode, CID spectrum of the precursor ion at *m/z* 433.221. (b) Positive-ion mode, CID spectrum of the precursor ion at *m/z* 417.226. (c) Positive-ion mode, CID spectrum of the precursor ion at *m/z* 401.199.

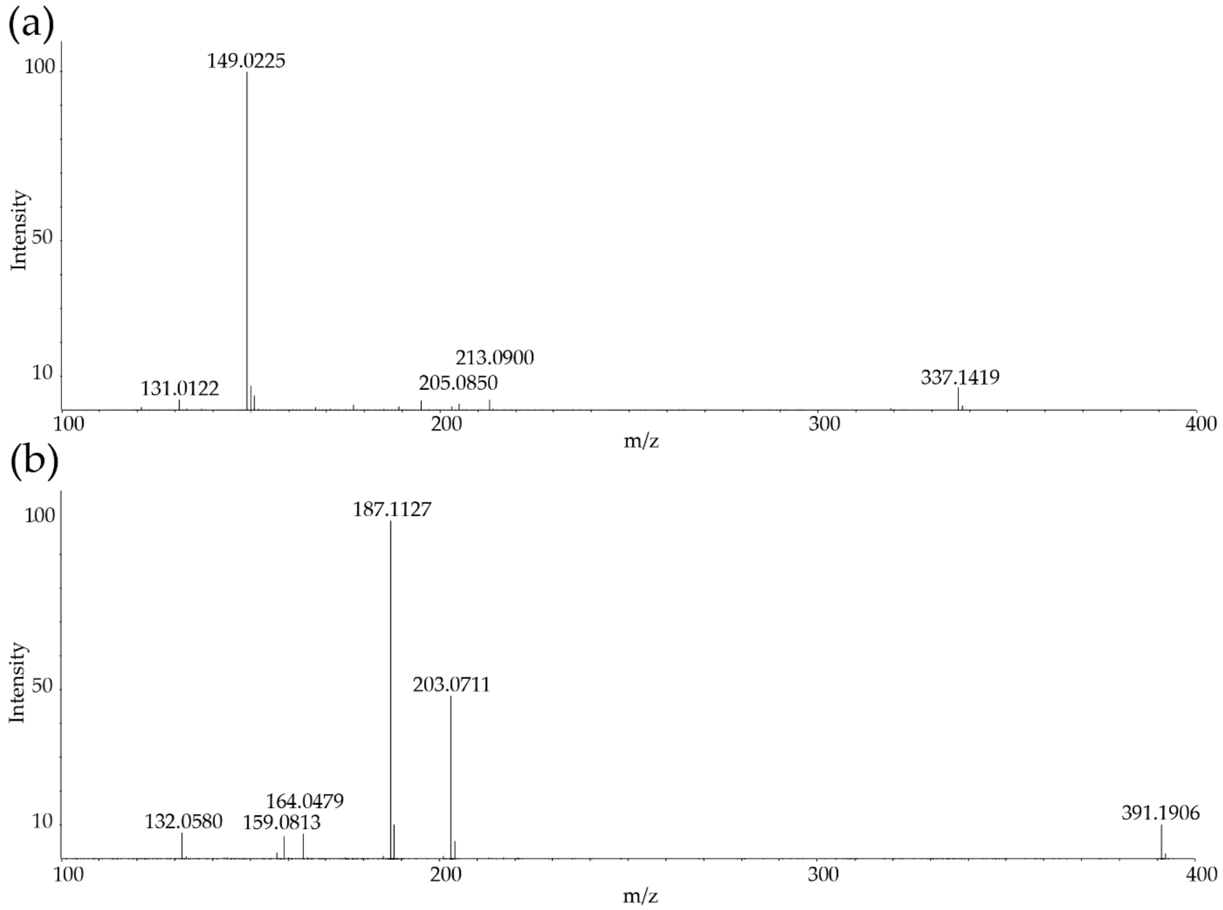

Figure S8. MS/MS fragmentation spectra for the compound isolated from *Astragalus dasyanthus*. (a) Positive-ion mode, CID spectrum of the precursor ion at *m/z* 393.204. (b) Positive-ion mode, CID spectrum of the precursor ion at *m/z* 391.191.
