# Supplementary figures and images for: HR-LCMS/MS-Based Dereplication of Plant-Derived Autophagy Inducers Revealed Astragalus dasyanthus as a New Glabrol Producer
Source: Metabolites. 2026 May 1;16(5):311. doi: 10.3390/metabo16050311 (PMC13208147; doi:10.3390/metabo16050311)

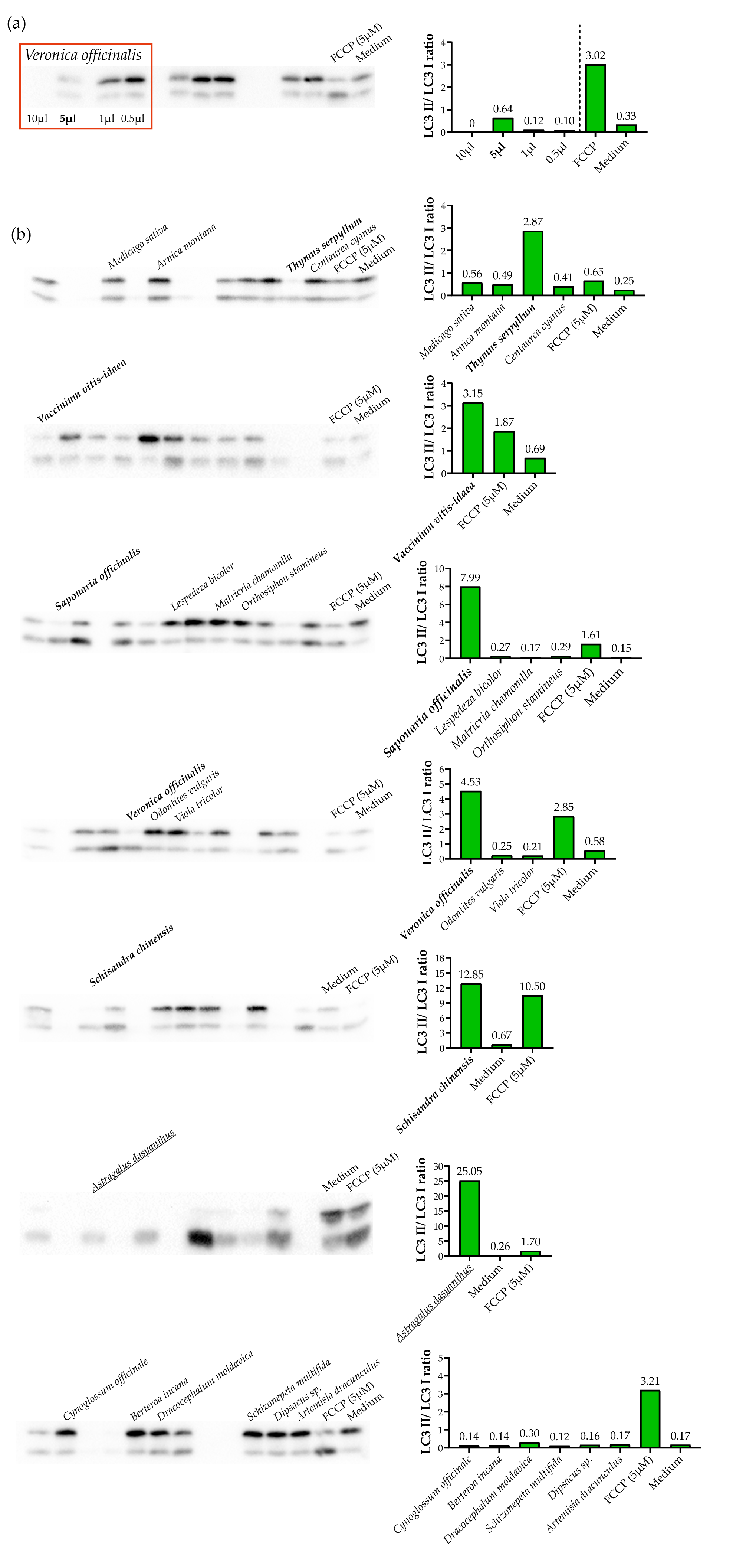

Supplement: Supplementary file 1 [file metabolites-16-00311-s001.zip › Figure S1.tif]

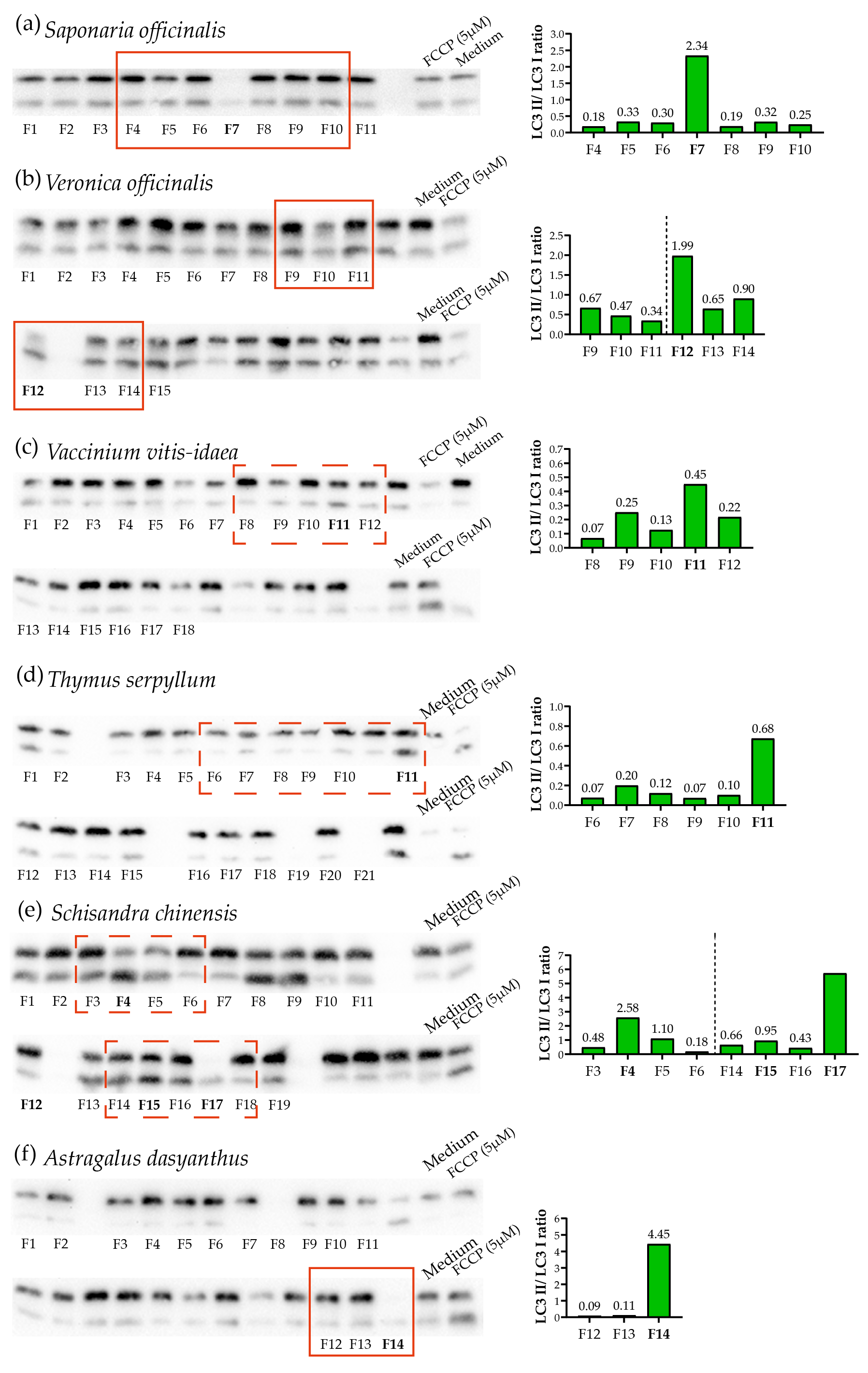

Supplement: Supplementary file 1 [file metabolites-16-00311-s001.zip › Figure S2.tif]

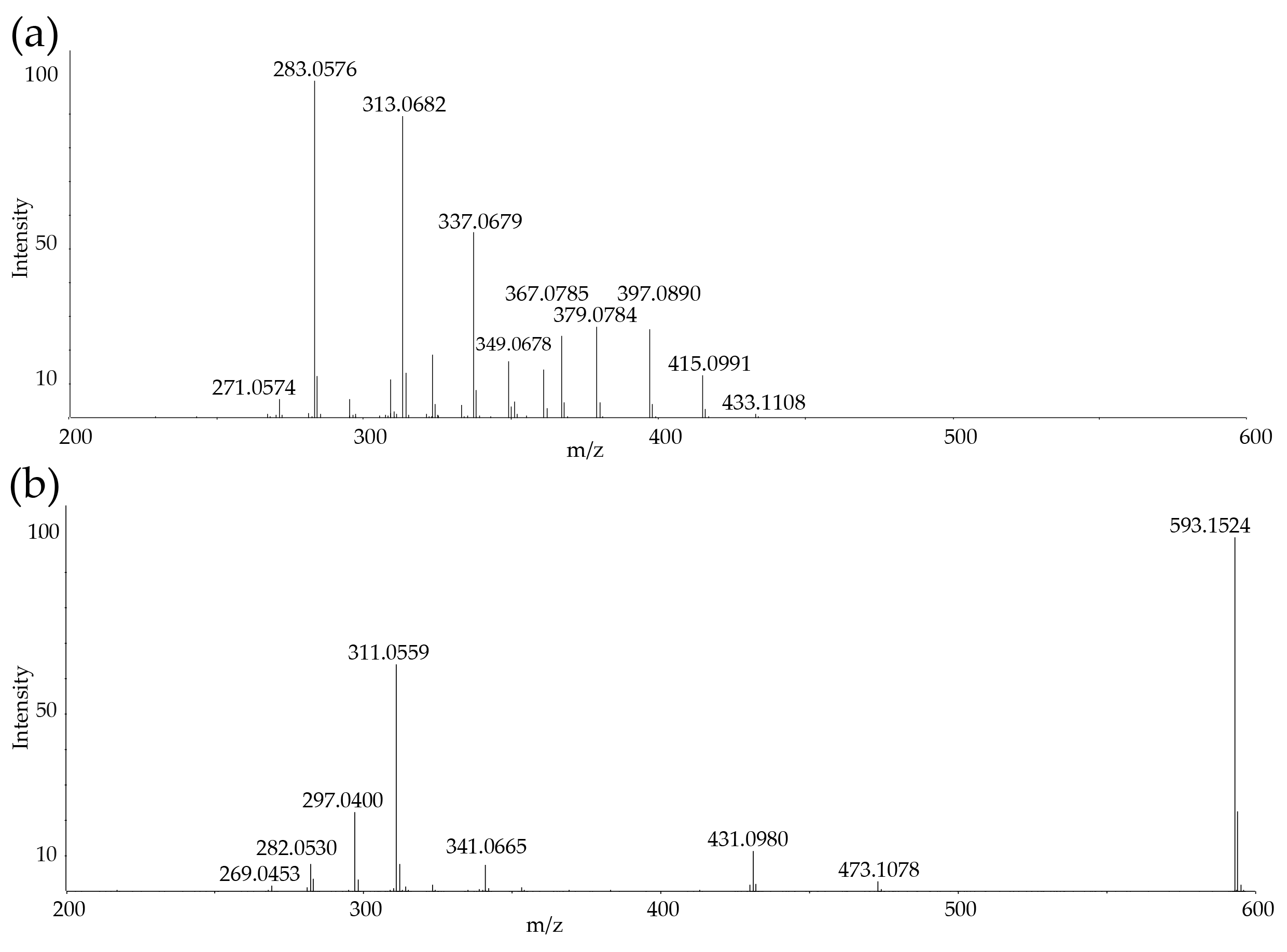

Supplement: Supplementary file 1 [file metabolites-16-00311-s001.zip › Figure S3 (Saponaria officinalis).tif]

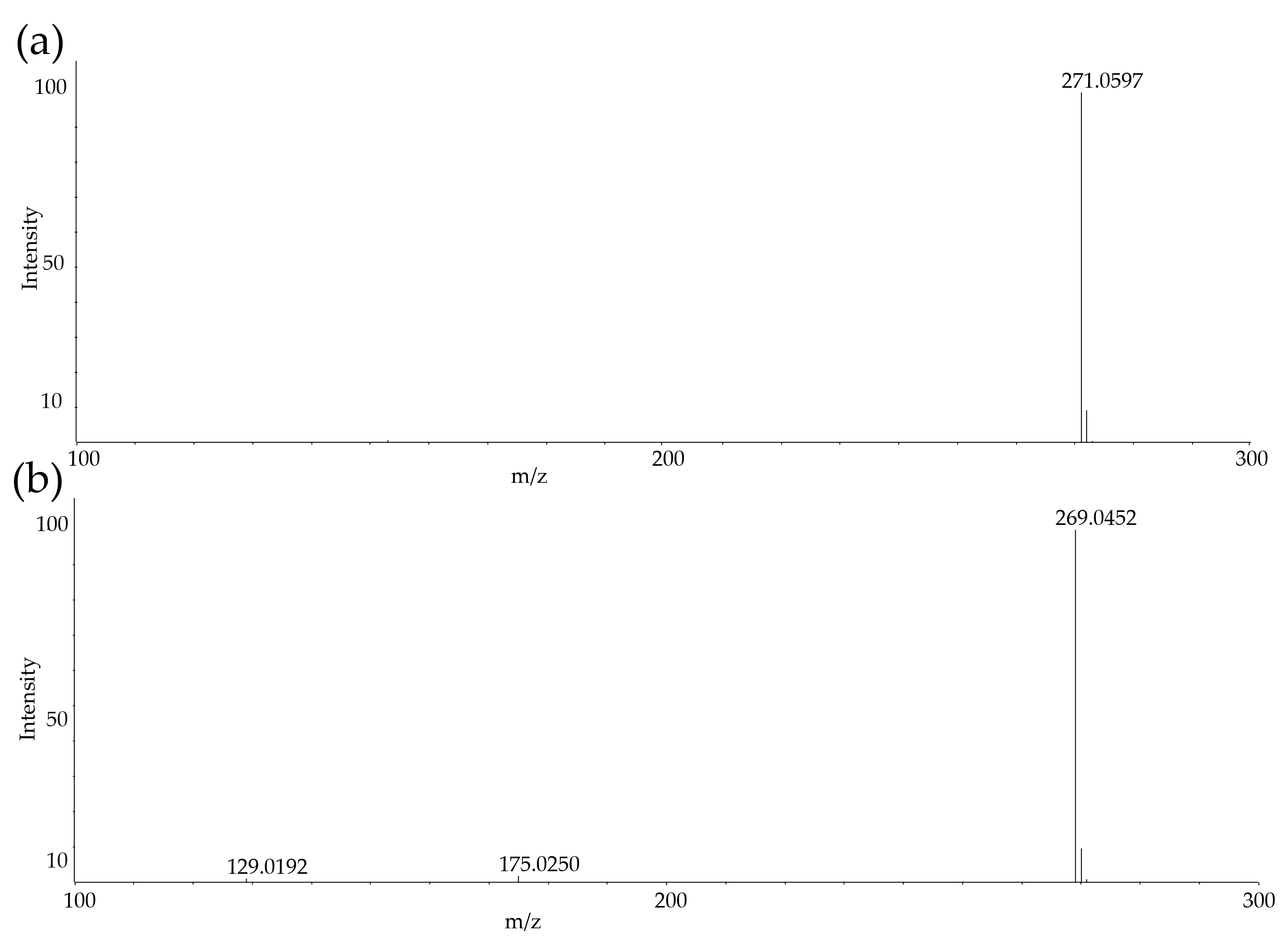

Supplement: Supplementary file 1 [file metabolites-16-00311-s001.zip › Figure S4 (Verónica officinalis).tif]

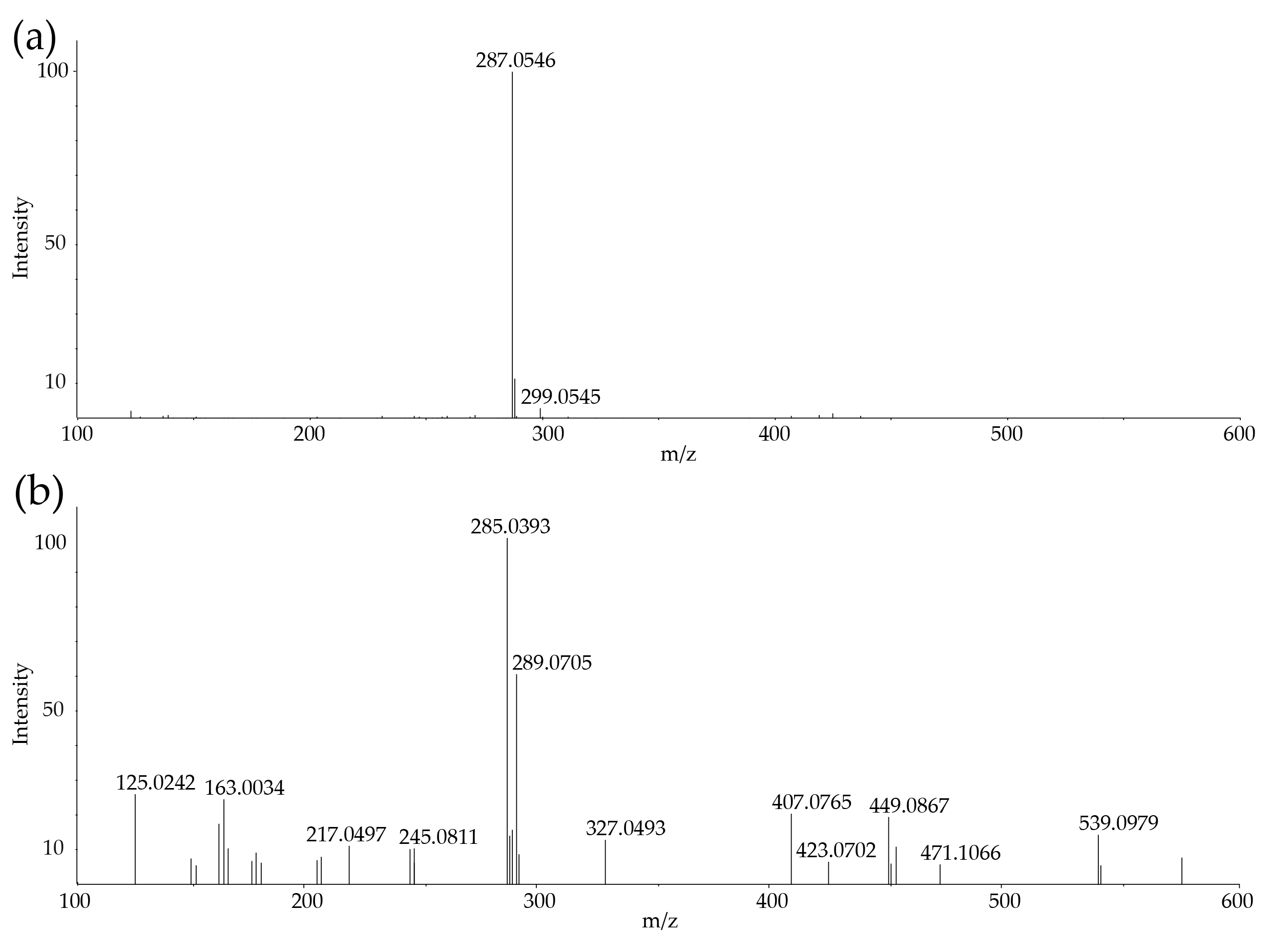

Supplement: Supplementary file 1 [file metabolites-16-00311-s001.zip › Figure S5 (Vaccinium vitis-idaea).tif]

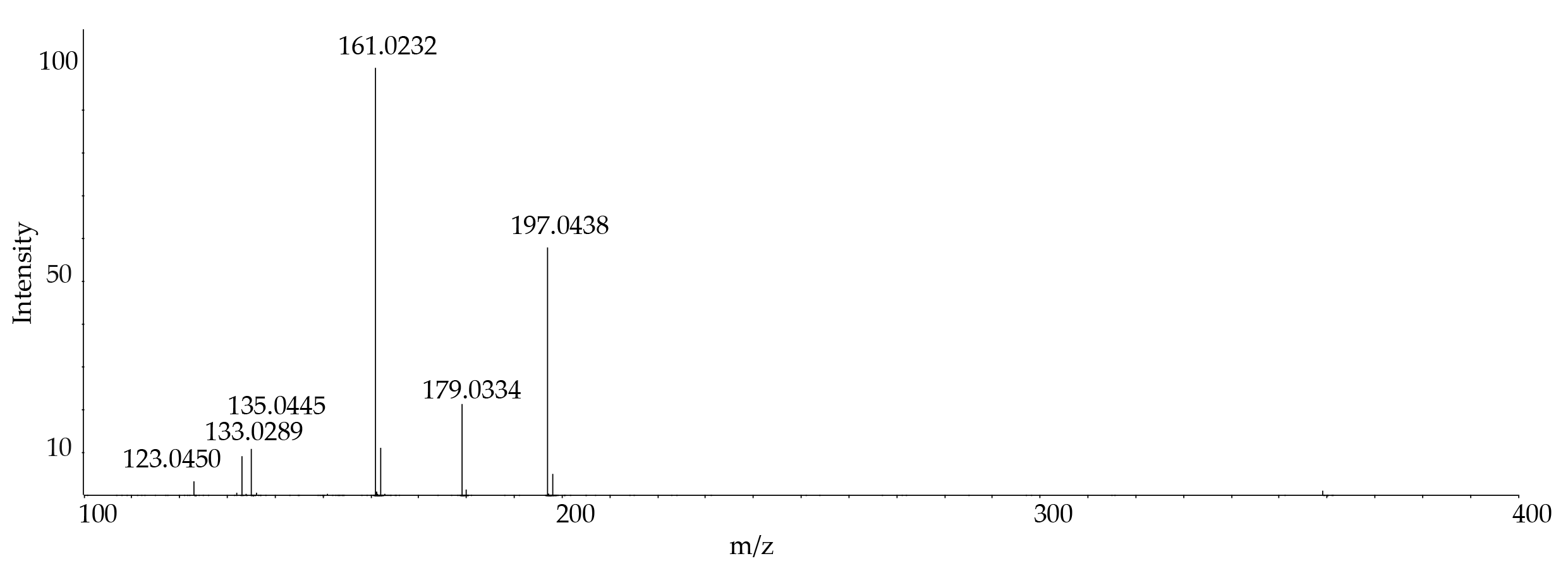

Supplement: Supplementary file 1 [file metabolites-16-00311-s001.zip › Figure S6 (Thymus serpyllum).tif]

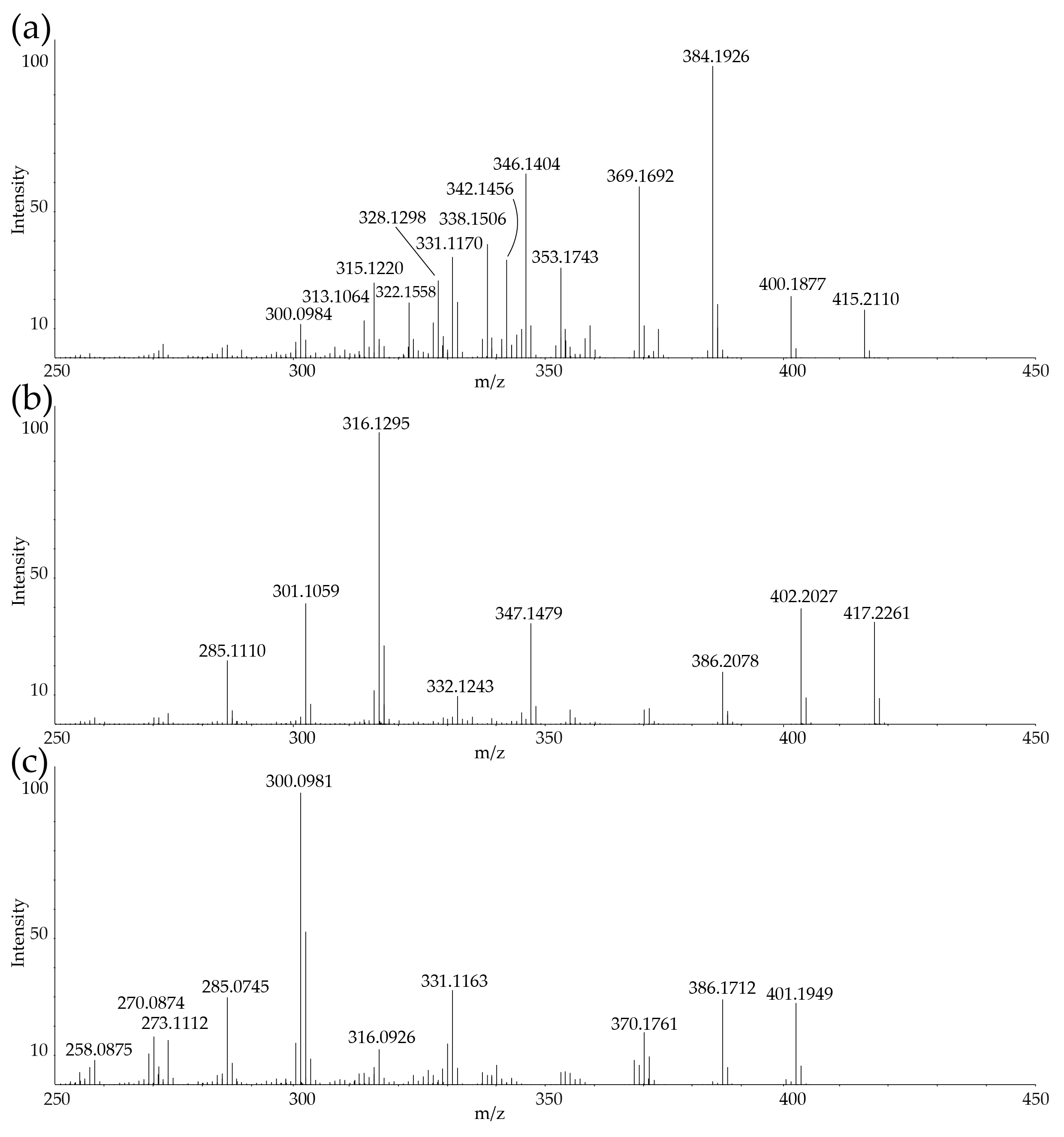

Supplement: Supplementary file 1 [file metabolites-16-00311-s001.zip › Figure S7 (Schisandra chinensis).tif]

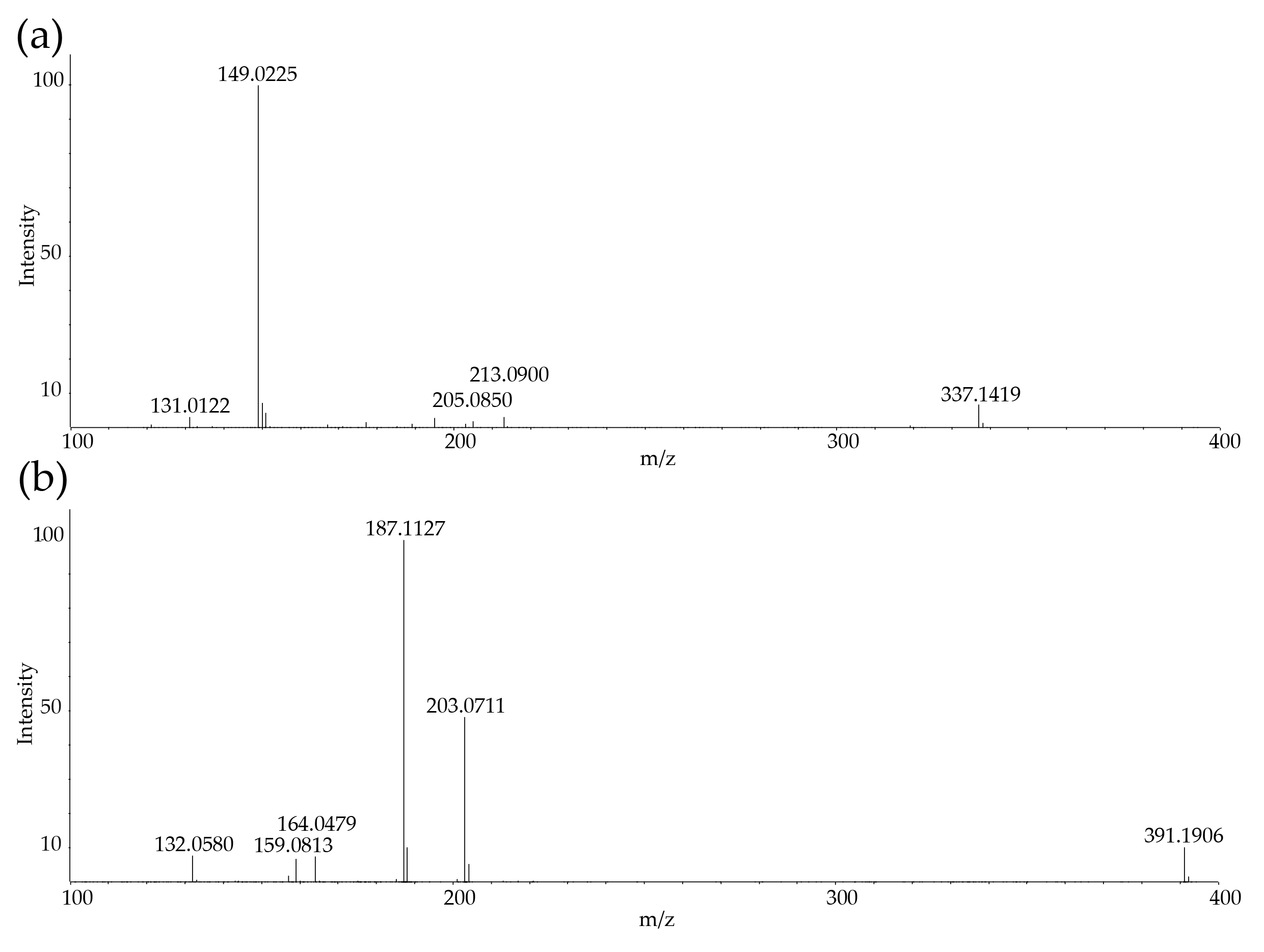

Supplement: Supplementary file 1 [file metabolites-16-00311-s001.zip › Figure S8 (Astragalus dasyanthus).tif]
